# Advanced Glycated apoA-IV Loses Its Ability to Prevent the LPS-Induced Reduction in Cholesterol Efflux-Related Gene Expression in Macrophages

**DOI:** 10.1155/2020/6515401

**Published:** 2020-01-14

**Authors:** Ligia Shimabukuro Okuda, Rodrigo Tallada Iborra, Paula Ramos Pinto, Ubiratan Fabres Machado, Maria Lucia Corrêa-Giannella, Russell Pickford, Tom Woods, Margaret Anne Brimble, Kerry-Anne Rye, Marisa Passarelli

**Affiliations:** ^1^Laboratório de Lípides (LIM 10), Hospital das Clínicas (HCFMUSP), Faculdade de Medicina da Universidade de São Paulo, São Paulo, Brazil; ^2^Universidade São Judas Tadeu, São Paulo, Brazil; ^3^Department of Physiology and Biophysics, Institute of Biomedical Sciences, University of São Paulo, São Paulo, Brazil; ^4^Laboratório de Carboidratos e Radioimunoensaio (LIM 18), Hospital das Clínicas (HCFMUSP), Faculdade de Medicina da Universidade de São Paulo, São Paulo, Brazil; ^5^Programa de Pós-Graduação em Medicina, Universidade Nove de Julho, São Paulo, Brazil; ^6^Bioanalytical Mass Spectrometry Facility, University of New South Wales, Sydney, Australia; ^7^School of Chemical Sciences, University of Auckland, Auckland, New Zealand; ^8^Lipid Research Group, School of Medical Sciences, University of New South Wales, Sydney, Australia

## Abstract

We addressed how advanced glycation (AGE) affects the ability of apoA-IV to impair inflammation and restore the expression of genes involved in cholesterol efflux in lipopolysaccharide- (LPS-) treated macrophages. Recombinant human apoA-IV was nonenzymatically glycated by incubation with glycolaldehyde (GAD), incubated with cholesterol-loaded bone marrow-derived macrophages (BMDMs), and then stimulated with LPS prior to measurement of proinflammatory cytokines by ELISA. Genes involved in cholesterol efflux were quantified by RT-qPCR, and cholesterol efflux was measured by liquid scintillation counting. Carboxymethyllysine (CML) and pyrraline (PYR) levels, determined by Liquid Chromatography-Mass Spectrometry (LC-MS/MS), were greater in AGE-modified apoA-IV (AGE-apoA-IV) compared to unmodified-apoA-IV. AGE-apoA-IV inhibited expression of interleukin 6 (*Il6*), TNF-alpha (*Tnf*), IL-1 beta (*Il1b*), toll-like receptor 4 (*Tlr4*), tumor necrosis factor receptor-associated factor 6 (*Traf6*), Janus kinase 2/signal transducer and activator of transcription 3 (*Jak2/Stat3*), nuclear factor kappa B (*Nfkb*), and AGE receptor 1 (*Ddost*) as well as IL-6 and TNF-alpha secretion. AGE-apoA-IV alone did not change cholesterol efflux or ABCA-1 levels but was unable to restore the LPS-induced reduction in expression of *Abca1* and *Abcg1*. AGE-apoA-IV inhibited inflammation but lost its ability to counteract the LPS-induced changes in expression of genes involved in macrophage cholesterol efflux that may contribute to atherosclerosis.

## 1. Introduction

The inverse relationship between plasma HDL-cholesterol (HDL-C) levels and cardiovascular disease (CVD) risk is well established in epidemiological studies [1]. Nonetheless, many clinical trials of agents that increase plasma HDL-C and Mendelian randomization studies have failed to demonstrate that increasing HDL-C levels reduces CV endpoints [2–4]. These negative outcomes have led to the conclusion that HDL functionality may be a better marker of the cardioprotective properties of HDL than measurement of plasma HDL-C or apoA-I levels [5, 6].

HDLs are heterogeneous particles varying in composition and size [7]. The major HDL apolipoprotein is apoA-I, followed by apoA-II and apoA-IV. ApoA-I and apoA-IV both inhibit inflammation, oxidation, platelet aggregation, and thrombosis [8–12]. ApoA-IV also modulates triglyceride-rich lipoprotein assembly and metabolism, improves glucose homeostasis, and inhibits food intake [13–19]. ApoA-IV additionally mediates steps of the reverse cholesterol transport (RCT) pathway, where excess cholesterol from the arterial wall is transported to the liver for excretion into bile and feces. This occurs by the interaction of lipid-free apolipoproteins and HDL to the ATP-binding cassette transporters A1 (ABCA-1) and G1 (ABCG-1), activation of lecithin cholesterol acyltransferase (LCAT), activity of cholesteryl ester transfer protein (CETP), and uptake of esterified cholesterol from HDL by the hepatic scavenger receptor B-I (SR-BI) [20–25].

Clinical studies in humans and HDL proteome data have demonstrated an inverse association between plasma apoA-IV levels and CVD [26, 27]. In addition, exogenous administration or overexpression of A-IV in apoE knockout mice reduces the atherosclerotic lesion area [8–10, 28, 29]. In this context, apoA-IV is considered a potential therapeutic target for the prevention or treatment of atherosclerosis [30].

Advanced glycation end products (AGEs) are prevalent in diabetes mellitus (DM) and impair HDL functionality [31, 32]. AGEs are generated when glucose and oxoaldehydes interact nonenzymatically with lysine and arginine residues in the amino terminal domain of proteins such as apolipoproteins. They are also generated by the nonenzymatic interaction of glucose and oxoaldehydes with phospholipids and nucleic acids [33]. AGE formation has also been reported in patients with inflammatory conditions and chronic kidney disease [34]. Exogenous sources of AGEs include food cooked at a high temperature and tobacco [33, 35].

Nonenzymatic glycation of apoA-I reduces HDL function as well as increases coronary artery plaque progression in patients with type 2 diabetes mellitus (T2DM) [36–41]. A recent study demonstrated that the extent of derivatization of apoA-IV by the AGE carboxymethyllysine (CML) is associated with coronary heart disease severity [42].

The present study is concerned with how the modification of apoA-IV by AGEs compromises its capacity to inhibit lipopolysaccharide- (LPS-) induced inflammation and macrophage cholesterol efflux.

## 2. Material and Methods

### 2.1. Production of Recombinant Human apoA-IV

A vector containing the apoA-IV gene (plasmid pET14b) was inserted into Clear-Coli BL21 (DE3) electrocompetent cells (Lucigen Corporation, Middleton, WI). The pET-14b vector contained an N-terminal histidine-tag sequence followed by a thrombin cleavage site and three cloning sites as well as the gene for ampicillin resistance. ApoA-IV expression was induced by addition of 1 M isopropyl-*β*-D-1-thiogalactopyranoside (IPTG). ApoA-IV was purified using 2 × 5 mL His-Trap fast flow columns connected in series to an AKTA FPLC system (GE Healthcare, Chicago, IL). The histidine-tag was removed by incubation for 24 h at 37°C with thrombin (2 U/mg protein). Endotoxin levels of the purified apolipoprotein were measured using a LAL chromogenic endotoxin quantitation kit (Thermo Fisher Scientific, Rockford, IL) and were ≤0.5 EU/mL [43].

### 2.2. Advanced Glycation of Recombinant apoA-IV

Recombinant apoA-IV was incubated for 24 h at 37°C without (unmodified-apoA-IV) or with 0.25, 0.5, or 1 mM glycolaldehyde (GAD; AGE-apoA-IV) (Sigma-Aldrich, St Louis, MO) in endotoxin-free phosphate buffer (PBS; NaCl 137 mmol/L; Na_2_HPO_4_ 4 mmol/L; KCl 2 mmol/L; K_2_PO_4_ 1 mmol/L, pH 7.4), then dialyzed against PBS and filtered (0.22 *μ*m filter). The final concentration of unmodified AGE-apoA-IV was determined using a BCA Protein Assay Kit (Pierce Biotechnology, Rockford, IL).

### 2.3. Liquid Chromatography-Mass Spectrometry (LC-MS/MS)

LC-MS/MS was used to determine carboxymethyllysine (CML) and pyrraline (PYR) levels in apoA-IV as previously described [44]. Briefly, unmodified and AGE-apoA-IV were concentrated and digested using four different enzymes (pepsin, pronase E, aminopeptidase, and prolidase). Samples were separated and analyzed by high-performance liquid chromatography (HPLC) coupled directly to a TSQ Quantum Access triple quadrupole mass spectrometer (Thermo Fisher Scientific, Bremen, Germany) via an electrospray interface operating in the positive mode. Calibration curves generated with a range of concentrations (0-10 ng of CML/*μ*L and 0-100 pg of PYR/*μ*L) of unlabeled standards and a constant concentration of isotopically labelled standards (ISTD) were used to calculate the amount of CML and PYR in samples.

### 2.4. Cell Culture

Animal care was performed in accordance to the National Research Council (US) Committee for the Update of the Guide for the Care and Use of Laboratory Animals (2011) and approved by Animal Care and Research Advisory Committee (Faculdade de Medicina da Universidade de Sao Paulo—CEUA 607/1302). Bone marrow-derived cells were isolated from wild-type C57BL/6 mice and cultured for 5 days at 37°C under 5% CO_2_ in L929 cell-conditioning media (NCTC clone 929, code 0188, Cell Bank Rio de Janeiro—BCRJ) containing colony stimulating factor-1 (CSF-1) [45]. Differentiated bone marrow-derived macrophages (BMDMs) were loaded with cholesterol by incubation for 24 h with low-glucose Dulbecco's modified Eagle Medium (DMEM) and acetylated LDL (50 *μ*g of protein/mL) [46]. Unmodified or AGE-apoA-IV (glycated with 1 mM GAD) were then added to the cells at a final concentration of 50 *μ*g/mL. After a further 48 h of incubation, the apoA-IV was removed, and the macrophages were incubated for 24 h with lipopolysaccharide (LPS) (1 *μ*g/mL; *E. coli* serotype 026:B6; Sigma-Aldrich).

The medium was then removed, centrifuged for 10 min at 453 x*g* and frozen (-20°C) until use. The concentrations of interleukin 6 (IL-6) and tumor necrosis factor-alpha (TNF-alpha) were determined by ELISA (R&D System, DuoSet, Minneapolis, MN).

### 2.5. Cholesterol Efflux Assay

BMDMs were loaded with cholesterol by incubation for 48 h with acetylated LDL (50 *μ*g of protein/mL) and ^14^C-cholesterol (0.3 uCi/mL, Perkin Elmer, Boston, MA) then incubated with DMEM containing fatty acid-free bovine serum albumin (BSA) for 24 h to equilibrate cell cholesterol pools. The cells were then incubated for 8 h with unmodified-apoA-IV or AGE-apoA-IV (30 *μ*g/mL). Radioactivity was measured in the medium and in the cells. The % cholesterol efflux was calculated as described by Machado et al. [47].

### 2.6. Analysis of Gene Expression

RNA was isolated following the protocol as described by the manufacturer (RNeasy Mini Kit, Qiagen, Hilden, Germany). Sample integrity and concentration were evaluated using an Agilent Bioanalyzer RNA Kit (Agilent, Santa Clara, CA). cDNA was synthesized from 200 ng of total RNA using High-Capacity RNA-to-cDNA Kit (Applied Biosystems, Foster City, CA) then stored at -20°C until use. A StepOne Real Time PCR System and Gene Expression Master Mix (Applied Biosystems) were used for RT-qPCR analysis. The following probes were used (TaqMan): *Tnf*: Mm00450234_m1, Il6: Mm00450234_m1, *Il1b*: Mm00434228_m1, *Ccl2:* Mm00441242_m1, *Tlr4:* Mm00445273_m1, *Myd88:* Mm00440338_m1, *Traf6*: Mm00493836_m1, *Nfkb*: Mm00481872_s1, *Rela*: Mm00501346_m1, *Jak2*: Mm01208489_m1, *Stat3*: Mm01219775_m1, *Ager*: Mm01134790_g1, *Ddost*: Mm00492100_m1, *Abca1*: Mm00442646_m1, *Abcg1*: Mm00437390_m1, *Scarb1*: Mm00450234_m1, *Nr1h3*: Mm01329744_g1, and *Nr1h2*: Mm00437265_g1. The relative expression of each gene was normalized to *β*-actin (Actb: Mm00607939_s1). Relative quantification of gene expression was performed using StepOne software 2.0 (Applied Biosystems) and the comparative method of cycle threshold (Ct) (2-*Δ*ΔCt) [48].

### 2.7. Western Blotting

Cholesterol-loaded BMDMs were pretreated with unmodified or AGE-apoA-IV and then incubated with LPS as described above. Cells were then washed with cold PBS, centrifuged (14000 rpm, 5 min, 4°C) and resuspended in Tris buffered saline (TBS) containing protease inhibitors and preservatives (1.5 mM aprotinin, 2 *μ*M pepstatin, 1 *μ*M leupeptin, and 0.1 mM PMSF). The cells were lysed by sonication (12 sec, 5% amplitude—Branson Sonifier 450, Danbury, CT, USA). The cell membrane fraction was isolated by centrifugation (15 min, 14000 rpm, 4°C), and the protein concentration was determined by the BCA method (Pierce Biotechnology, Rockford, IL). The samples (50 *μ*g) were loaded onto a polyacrylamide gel (6.5%), electrophoresed for 1.5 h, transferred to a PVDF membrane, and immunoblotted with an anti-ABCA1 monoclonal antibody (1 : 50; MABI98-7). The monoclonal antibody to mouse and human ABCA1 (MABI98-7) was generated in rats against the C-terminal peptide CNFAKDQSDDDHLKDLSLHKN and was kindly provided by Professor Shinji Yokoyama (MAB Institute, Japan). Bands were visualized by ECL (WESTAR, Cyanagen, Bologna, Italy). Image capture was performed using ImageQuant 350 (GE Healthcare). Results were expressed in arbitrary units and normalized to beta-actin (Actin-pan antibody 10R-A106A; 1 : 1000, Fitzgerald Industries Int., Acton, MA, USA).

### 2.8. Statistical Analysis

Statistical analysis was performed using GraphPad Prism 6.05 (GraphPad Software, Inc.). One way ANOVA with Tukey's post-test or Student's *t* tests were used to compare groups. All situations with a descriptive level of significance of <5% were considered as significant.

## 3. Results

### 3.1. Pyralline and Carboxymethyllysine Content in Glycolaldehyde Modified apoA-IV

**Figure 1:**
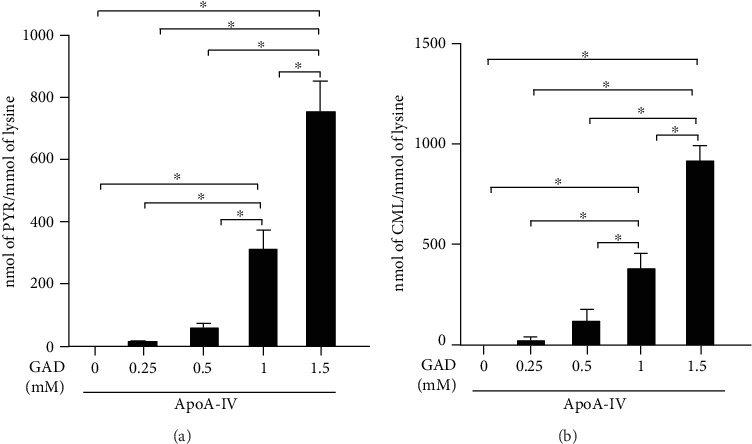
Pyrraline (PYR) and carboxymethyllysine (CML) content in ApoA-IV submitted to *in vitro* advanced glycation. ApoA-IV was incubated for 24 h, at 37°C with different concentrations of glycolaldehyde (GAD). After digestion, samples were analyzed by LC-MS/MS (*n* = 9). One-way ANOVA with Tukey's post-test was utilized to compare groups (data from 3 independent experiments; mean ± SD; ^∗^*p* < 0.0001).

ApoA-IV was incubated with increasing concentrations of GAD (0.25, 0.5, and 1 mM), and AGE formation was assessed by LC-MS/MS. As shown in Figure 1, there was a dose-dependent generation of PYR (Figure 1(a)) and CML (Figure 1(b)) in apoA-IV.

### 3.2. AGE-apoA-IV Maintains Its Ability to Inhibit Inflammation but Is Less Efficient than Unmodified-apoA-IV

**Figure 2:**
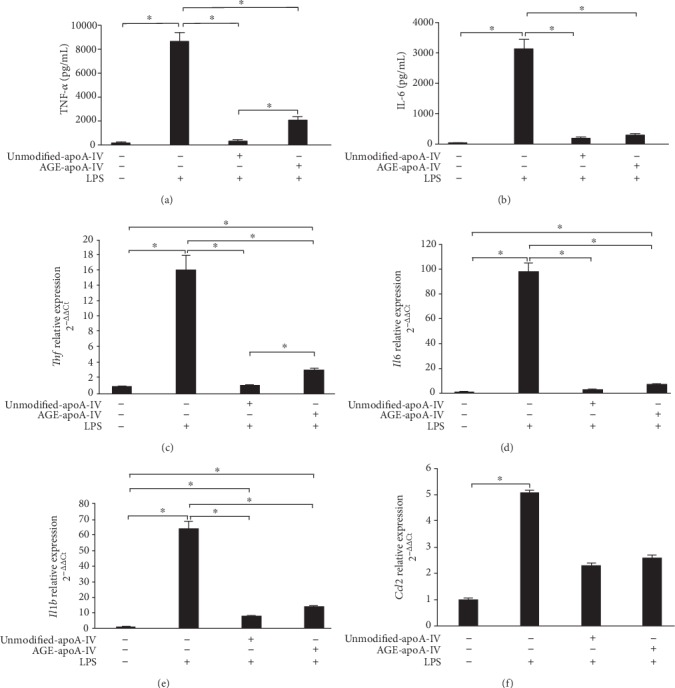
TNF-alpha and IL-6 secretion and *Tnf*, *Il6*, *Il1b*, and *Ccl2* mRNA expression by macrophages treated with unmodified or AGE-apoA-IV and further stimulated with LPS. Bone marrow-derived macrophages (BMDMs) were overloaded with acetylated LDL (50 *μ*g/mL) and incubated with unmodified or AGE-apoA-IV (*n* = 9). Control incubations were kept in DMEM/FAFA alone. After washing, macrophages were stimulated for 24 h with 1 *μ*g/mL of LPS. TNF-alpha and IL-6 secretion were quantified by ELISA (a and b). Gene expression (c–f) was determined by RT-qPCR as described in Material and Methods section. Data are representative of 3 independent experiments (mean ± SD; ^∗^*p* < 0.05).

The ability of AGE-apoA-IV to inhibit macrophage inflammation elicited by LPS was investigated. Incubation with LPS increased the secretion of TNF-alpha and IL-6 in BMDMs by 56-fold (Figure 2(a)) and 123-fold (Figure 2(b)), respectively. Preincubation of the BMDMs with unmodified-apoA-IV inhibited the increase in TNF-alpha and IL-6 secretion by 96 ± 0.9% and 94 ± 0.8%, respectively. TNF-alpha secretion was inhibited by 76 ± 10% in BMDMs that were preincubated with AGE-apoA-IV, although this effect was less compared to unmodified-apoA-IV. AGE-apoA-IV inhibited IL-6 secretion as effectively as unmodified-apoA-IV. These results indicate that advanced glycation minimally impairs the anti-inflammatory properties of apoA-IV. This was confirmed by measuring mRNA levels of *Tnf* (Figure 2(c)) and *Il6* (Figure 2(d)).

Incubation with LPS also increased interleukin-1 beta (*Il1b*) (Figure 2(e)) and *Ccl2* (that encodes the chemokine c-c motif ligand 2/monocyte chemotactic protein; MCP-1) mRNA levels (Figure 2(f)), 64-fold and 5-fold, respectively. The LPS-mediated increase in *Il1b* was reduced by 87 ± 7.2% in BMDMs that were preincubated with unmodified-apoA-IV and by 73 ± 17.5% in BMDMs that were preincubated with AGE-apoA-IV (Figure 2(e)). No changes were observed in the expression of *Ccl2* (Figure 2(f)).

**Figure 3:**
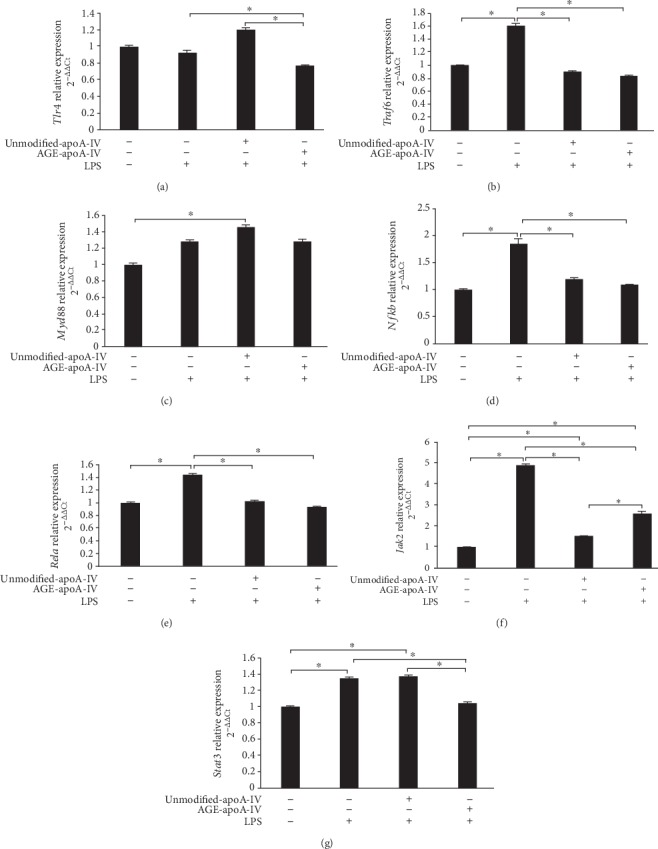
*Tlr4*, *Traf6*, *Myd88*, *Nfkb*, *Rela*, *Jak2*, *and Stat3* expression by macrophages treated with unmodified or AGE-apoA-IV and further stimulated with LPS. Bone marrow-derived macrophages (BMDMs) were overloaded with acetylated LDL (50 *μ*g/mL) and incubated with unmodified or AGE-apoA-IV. After washing, macrophages were stimulated for 24 h with 1 *μ*g/mL of LPS (*n* = 9). Gene expression was determined by RT-qPCR as described in Material and Methods section. Data are representative of 3 independent experiments (mean ± SD, ^∗^*p* < 0.05).

LPS interacts with TLR-4 at the macrophage cell surface, triggering increased expression of the myeloid differentiation primary response gene 88 (Myd88) and tumor necrosis factor receptor-associated factor 6 (Traf6) signaling that leads to the production of inflammatory mediators. *Tlr4* mRNA levels were not changed in cells incubated with LPS alone or preincubated with unmodified-apoA-IV prior to LPS. AGE-apoA-IV reduced *Tlr4* mRNA levels by 16 ± 13% in macrophages that were incubated with LPS as compared to unmodified-apoA-IV (Figure 3(a), *p* < 0.05). Incubation with LPS increased *Traf6* mRNA levels by 1.6-fold compared to control BMDMs (Figure 3(b)). Preincubation of BMDMs with unmodified or AGE-apoA-IV reduced the LPS-mediated increase in *Traf6* expression by 41 ± 17% and 44 ± 24%, respectively (Figure 3(b)). *Myd88* mRNA levels were surprisingly increased by 50 ± 35% compared to control in BMDMs that were preincubated with unmodified-apoA-IV. Incubation with LPS or LPS plus AGE-apoA-IV did not affect BMDM *Myd88* mRNA levels (Figure 3(c)). mRNA levels of the final intracellular effectors of the inflammatory LPS signaling, *Nfkb* and *Rela*, were significantly increased in LPS-treated BMDMs. Cells that had been preincubated with unmodified or AGE-apo-IV reduced the LPS-elicited increase in *Nfkb* mRNA levels by 32 ± 26% and 41 ± 7%, respectively, (*p* < 0.05), while *Rela* mRNA levels were reduced by 27 ± 15% and 34 ± 12%, respectively, (*p* < 0.05) compared to cells incubated with LPS alone (Figures 3(d) and 3(e)).

LPS also activates the janus kinase 2/signal transducer and activator of transcription 3 (JAK2/STAT3) pathway. Incubation with LPS increased the *Jak2* mRNA level in BMDMs by 5-fold. *Jak2* mRNA levels were reduced by 69 ± 2% and 42 ± 26%, (*p* < 0.05) in BMDMs that were preincubated, respectively, with unmodified and AGE-apoA-IV (Figure 3(f)). Compared to control cells, incubation of BMDMs with LPS alone and unmodified-apoA-IV plus LPS increased *Stat3* mRNA levels by 1.3-fold and 1.4-fold, respectively (*p* < 0.05) (Figure 3(g)). On the other hand, preincubation with AGE-apoA-IV reduced *Stat3* mRNA levels in LPS-activated HMDMs by 21 ± 13% and 25 ± 12% as compared, respectively, to LPS and unmodified-apoA-IV plus LPS (*p* < 0.05) (Figure 3(g)). These results show that AGE-apoA-IV is able to inhibit the inflammatory signaling and proinflammatory cytokine production in LPS-stimulated BMDMs.

### 3.3. Unmodified and AGE-apoA-IV Reduce *Ddost* but Do Not Affect *Ager* Expression

**Figure 4:**
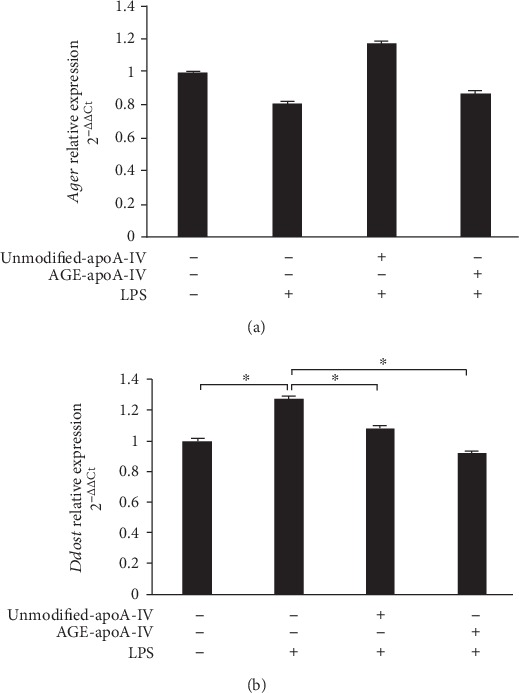
*Ager* and *Ddost* expression by macrophages treated with unmodified or AGE-apoA-IV and further stimulated with LPS. Bone marrow-derived macrophages (BMDMs) were overloaded with acetylated LDL (50 *μ*g/mL) and incubated with unmodified or AGE-apoA-IV. After washing, macrophages were stimulated for 24 h with 1 *μ*g/mL of LPS (*n* = 9). Gene expression was determined by RT-qPCR as described in Material and Methods section. Data are representative of 3 independent experiments (mean ± SD, ^∗^*p* < 0.05).

The mRNA level of the receptor for AGEs, RAGE (*Ager*), was unaffected by LPS or by preincubation with unmodified or AGE-apoA-IV (Figure 4(a)). On the other hand, *Ddost*, the gene that encodes AGER-1 (another AGE receptor that antagonizes RAGE signaling) was increased by 1.3-fold in BMDMs that had been incubated with LPS compared to control (*p* < 0.05) and downregulated by preincubation with unmodified-apoA-IV (13 ± 19%, *p* < 0.05) or AGE-apoA-IV (27 ± 11%, *p* < 0.05) compared to cells incubated with LPS alone (Figure 4(b)).

### 3.4. AGE-apoA-IV Does Not Affect ABCA-1 Protein Expression or Cholesterol Efflux

**Figure 5:**
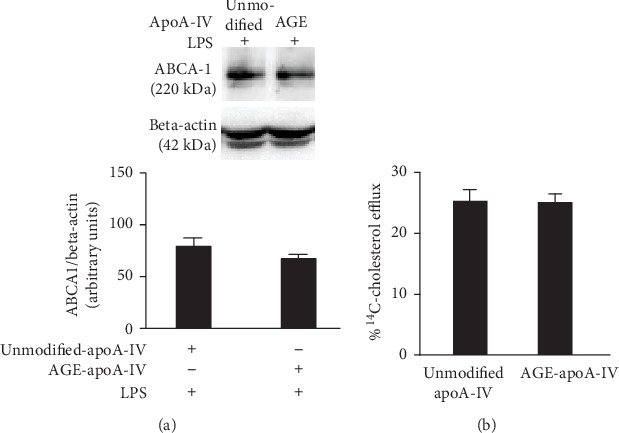
ABCA-1 expression and ^14^C-cholesterol efflux in macrophages treated with unmodified and AGE-apoA-IV (a). Bone marrow-derived macrophages (BMDM) were overloaded with acetylated LDL (50 *μ*g/mL) and incubated with unmodified or AGE-apoA-IV for 48 h. After washing, macrophages were stimulated for 24 h with 1 *μ*g/mL of LPS. The ABCA-1 protein level was determined by immunoblot (*n* = 3) (b). BMDMs were overloaded with acetylated LDL (50 *μ*g/mL) and ^14^C-cholesterol for 48 h. After maintenance in equilibrium medium, cells were incubated with unmodified or AGE-apoA-IV as cholesterol acceptors (*n* = 6). Cholesterol efflux was calculated as ^14^C-cholesterol in medium/^14^C-cholesterol in medium +^14^C-cholesterol in cells × 100. Basal efflux was determined in incubations with DMEM containing fatty acid-free albumin that was subtracted from those with apoA-IV. Data are representative of 3 independent experiments (mean ± SD) and were compared by the Student *t* test.

As shown in Figure 5(a), BMDM ABCA-1 protein levels were not affected by incubation with unmodified-apoA-IV or AGE-apoA-IV. In addition, the advanced glycation of apoA-IV did not change its ability in accepting excess cholesterol (Figure 5(b)).

### 3.5. AGE-apoA-IV Is Unable to Prevent the Reduction in *Abca1* and *Abcg1* mRNA Induced by LPS

**Figure 6:**
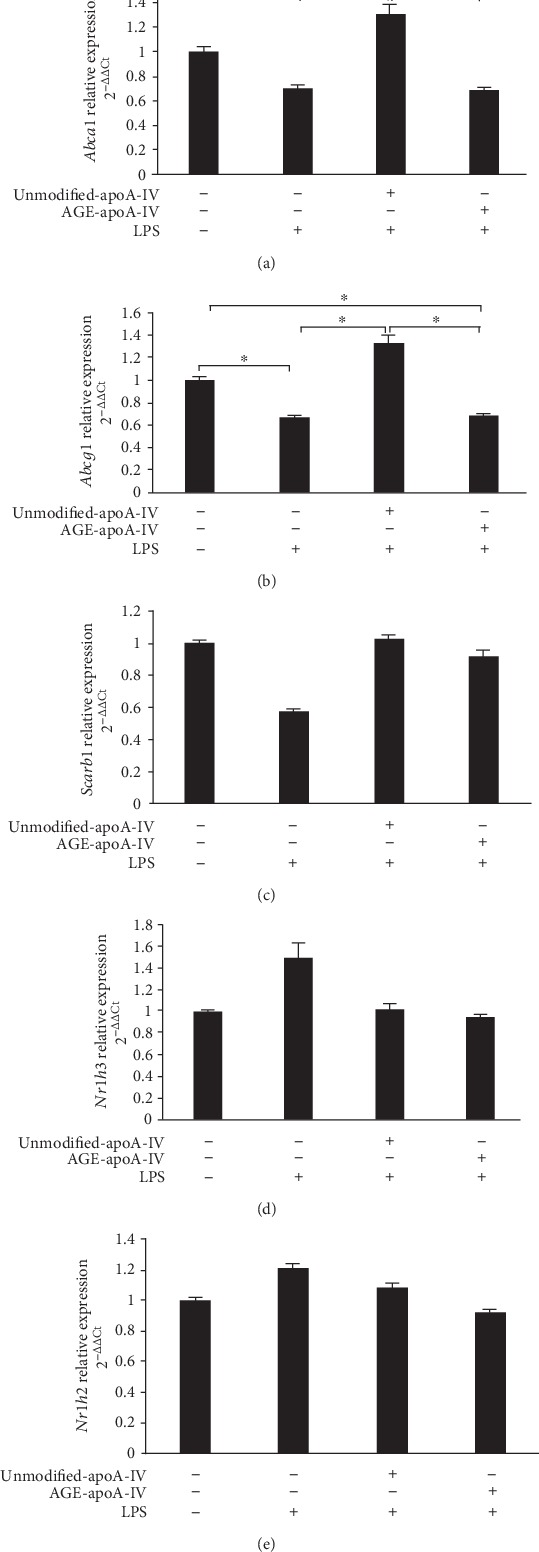
*Abca1*, *Abcg1*, *Scarb1*, *Nr1h3*, and *Nr1h2* mRNA expression in macrophages treated with unmodified or AGE-apoA-IV and further stimulated with LPS. Bone marrow-derived macrophages (BMDMs) were overloaded with acetylated LDL (50 *μ*g/mL) and incubated with unmodified or AGE-apoA-IV. After washing, macrophages were stimulated for 24 h with 1 *μ*g/mL of LPS (*n* = 9). Gene expression was determined by RT-qPCR as described in Material and Methods section. Data are representative of 3 independent experiments (mean ± SD, ^∗^*p* < 0.05) and were compared by ANOVA and Tukey's post-test.

Incubation with LPS reduced *Abca1* and *Abcg1* mRNA levels by 28 ± 19% and 32 ± 16%, respectively (Figures 6(a) and 6(b)). This reduction in *Abca1* and *Abcg1* mRNA was prevented by preincubation of the cells with unmodified-apoA-IV, but not with AGE-apoA-IV (Figures 6(a) and 6(b)). No changes were observed in the *Scarb1* (SR-BI) gene expression elicited by LPS, and this was equally observed in incubations with unmodified and modified apoA-IV, respectively (Figure 6(c)). LXR alpha and beta, that are encoded by *Nr1h3* and *Nr1h2*, and control *Abca1* and *Abcg1* gene transcription were unaltered by LPS or unmodified and AGE-apoA-IV (Figures 6(d) and 6(e)).

## 4. Discussion

Chemical alterations of HDL apolipoproteins, including their nonenzymatic glycation by reactive *α*-oxoaldehydes such as methylglyoxal, glycolaldehyde, and glyoxal, are detrimental to their function and role in atheroprotection [36, 38, 40, 41]. Advanced glycation is independently related to cardiovascular disease, by inducing oxidative and inflammatory stress and disturbing the flux of cholesterol from the artery wall to the liver in the reverse cholesterol transport pathway. This may contribute to atherogenesis in diabetes mellitus (DM), chronic kidney disease, and inflammatory diseases [49–52].

In this study, it was investigated the effect of advanced glycation of apoA-IV on LPS-induced inflammation and the expression of genes that modulate cholesterol efflux in macrophages. It has been shown in macrophages that LPS represses *Abca1*, *Abcg1*, and *Scarb1* gene expression [53–55]. It is postulated that the effect of LPS on *Abca1* is based on NF-*κ*B activation that induces proinflammatory cytokine secretion and the expression of miR33 that represses the expression of *Abca1* and subsequently diminishes cholesterol efflux [54].

The production of proinflammatory mediators associated with cell injury induced by LPS involves the activation of the TLR-4/Myd88/Traf6 signal transduction pathway and the downstream effector NF-*κ*B, as well as the JAK/STAT and MAPK pathways [56–58]. In agreement with previous studies [8, 9, 22], our observations confirmed that unmodified-apoA-IV removes cholesterol from BMDMs and alleviates the LPS-induced inflammatory burst. This leads to diminished expression and secretion of proinflammatory cytokines as well as genes involved in LPS signaling such as *Tlr4*, *Traf6*, *Jak2*, *Stat3*, *Nfkb*, and *Rela*, while prevents the reduction induced by LPS in the expression *Abca1* and *Abcg1* genes.

Interestingly, AGE-apoA-IV significantly inhibited proinflammatory cytokine secretion and reduced mRNA levels of TNF-alpha and IL-6 in LPS-treated cells, nonetheless, less effectively than unmodified-apoA-IV. This is in contrast to the findings of Dai et al. (2017) [39], who reported increased expression and secretion of proinflammatory cytokines in human aortic endothelial cells (HAECs) treated with AGE-apoA-IV. These authors also showed that nonenzymatically glycated apoA-IV enhanced atherosclerotic lesion development in apoE knockout mice. These conflicting results may be related to the fact that Dai *et al*. (2017) [39] used concentration of 20 mM glyoxal to modify apoA-IV compared to 1 mM glycolaldehyde in the present study.

Although levels of proteins involved in TLR-4 signaling were not addressed by immunoblot in the present investigation, the expression of *Tlr4*, *Traf6*, *Nfkb*, *Rela*, *Jak2*, and *Stat3* genes was significantly reduced by AGE-apoA-IV in cells challenged by LPS.

The ability of glycated apoA-IV by itself to efflux cholesterol from macrophages was conserved. This is consistent with what has been reported previously for the efflux of cholesterol by nonenzymatically glycated apoA-I from the J774 macrophage cell line [59], but is contrary to others that showed reduction in cholesterol efflux mediated by glycated apoA-I in THP-1 and J774 macrophages [36, 38, 60].

Although preserving its ability in reducing the LPS-elicited inflammation, AGE-apoA-IV was unable to prevent the reduction in *Abca1* and *Abcg1* gene expression as compared to unmodified-apoA-IV. The exact mechanisms by which this effect occurs were not demonstrated at this point and require further investigation. Since *Abca1* and *Abcg1* mRNAs were reduced in cells incubated with LPS alone or with LPS plus AGE-apoA-IV, it is possible to consider that cholesterol efflux will be reduced by compromising the final amount of ABC protein.

CML is the major AGE formed *in vivo*, and it is present in atherosclerotic lesions [61]. Although different types of AGEs seem to bind to different domains of RAGE, which can interfere with their receptor affinity, CML binds to the V domain, while PYR, argpyrimidine, and pentosidine interact with the C domain [62, 63]. However, studies that have evaluated the effect of the interaction of specific AGEs with RAGE are still contradictory and limited to CML. While some investigators have shown that CML is an excellent RAGE ligand that increases inflammatory stress [64, 65], others have suggested the opposite [66, 67]. Additional studies are needed to identify and evaluate the affinity of different types of AGEs with RAGE and the resulting physiopathology.

*Ddost* encodes for AGER1, an AGE receptor whose expression is induced during glycoxidative and inflammatory stress allowing antagonism to the deleterious signaling of the AGE-RAGE axis [68]. In this investigation, we found reduction in *Ddost* mRNA levels in cells preincubated with unmodified and AGE-apoA-IV prior to LPS. This may be likely related to the capacity of both unmodified and glycated apoA-IV in inhibiting the inflammation elicited by LPS.

The current results are consistent with previous data from our group for isolated HDL [69]. Preincubation with HDL minimized the inflammatory effects of LPS in macrophages, although the exact mechanisms that are elicited by HDL or apo-IV, despite not being present during the LPS treatment, are not completely understood and worthy of further investigation.

## 5. Conclusion

In conclusion, although maintaining its anti-inflammatory activity, AGE-apoA-IV loses its ability in counteracting LPS effects on *Abca1* and *Abcg1* genes. This may contribute to atherogenesis in DM and other metabolic conditions that are associated with increased carbonyl stress. Future studies dealing with animal models susceptible to atherosclerosis treated with unmodified and AGE-apoA-IV will help to understand the antiatherogenic role of this apolipoprotein in the context of diabetes mellitus and glycoxidative stress.

## Data Availability

No data were used to support this study.
